# Assessing the reliability of gene expression measurements in very-low-numbers of human monocyte-derived macrophages

**DOI:** 10.1038/s41598-019-54500-8

**Published:** 2019-11-29

**Authors:** Carsten Geiß, Gregorio Alanis-Lobato, Miguel Andrade-Navarro, Anne Régnier-Vigouroux

**Affiliations:** 1 Institute of Developmental Biology and Neurobiology, Faculty of Biology, Johannes Gutenberg University of Mainz, Johann-Joachim-Becher-Weg 13, 55128 Mainz, Germany; 2 Human Embryo and Stem Cell Laboratory, The Francis Crick Institute, 1 Midland Road, London, NW1 1AT UK; 3 Institute of Organismic and Molecular Evolution, Faculty of Biology, Johannes Gutenberg University of Mainz, Hans-Dieter-Hüsch-Weg 15, 55128 Mainz, Germany

**Keywords:** Gene expression analysis, CNS cancer, Gene ontology

## Abstract

Tumor-derived primary cells are essential for *in vitro* and *in vivo* studies of tumor biology. The scarcity of this cellular material limits the feasibility of experiments or analyses and hence hinders basic and clinical research progress. We set out to determine the minimum number of cells that can be analyzed with standard laboratory equipment and that leads to reliable results, unbiased by cell number. A proof-of-principle study was conducted with primary human monocyte-derived macrophages, seeded in decreasing number and constant cell density. Gene expression of cells stimulated to acquire opposite inflammatory states was analyzed by quantitative PCR. Statistical analysis indicated the lack of significant difference in the expression profile of cells cultured at the highest (100,000 cells) and lowest numbers (3,610 cells) tested. Gene Ontology, pathway enrichment and network analysis confirmed the reliability of the data obtained with the lowest cell number. This statistical and computational analysis of gene expression profiles indicates that low cell number analysis is as dependable and informative as the analysis of a larger cell number. Our work demonstrates that it is possible to employ samples with a scarce number of cells in experimental studies and encourages the application of this approach on other cell types.

## Introduction

The use of primary cells in basic and clinical research is of upmost importance and interest because it facilitates the analysis of a biological material whose physiological properties (e.g. morphology, phenotype, function) are much less compromised than those of established, immortalized cell lines^1^. However, experimenting with these cells entails some restrictions due to (i) their properties (e.g. the (epi)genetic uniqueness of each donor), (ii) technical (e.g. differences resulting from the preparation of each sample) and (iii) practical issues (e.g. limited amount of material). This is a recurrent question in cancer research where researchers must deal not only with the high heterogeneity of the biological tissues of interest but also with problems associated with their physical availability and accessibility. This is the case, for instance, with the cellular material that can be obtained from biopsies of glioblastoma. Those brain tumors are characterized by a high level of molecular and cellular heterogeneity, which largely contribute to their resistance to therapy^2,3^. Tumor-associated microglia/macrophages (TAMs) constitute one subpopulation of glioblastoma cells that efficiently support tumor growth^4,5^ and, as such, represent attractive therapeutic targets^6^. Targeting these cells for therapeutic purposes necessitates a thorough knowledge of their properties. This knowledge has tremendously increased in the last few years thanks to experimental work performed with human primary microglia/macrophages^7–14^. However, the study of these highly plastic cells still poses experimental challenges. For instance, the patient to patient variability in terms of amount and quality of cells isolated from glioblastoma resections limits the extent and range of assays. Furthermore, discrete cellular phenotypic or functional profiles might vary with the location of the cells in the tumor or with their origin (resident microglia versus infiltrating macrophages). This heterogeneity and its biological significance typically cancel out in the analysis of cells pooled from the whole tumor. Two approaches might be considered to circumvent these limitations: the use of single-cell analysis or the use of a very low number of cells. Both would have to comply with the requirement of full reliability in the data generated by each setup.

Single cell profiling, namely single cell-RNA sequencing, combined with bioinformatics, is becoming a mainstream methodology to characterize the transcriptome of individual cells. A recent publication has reported profiles of TAMs freshly isolated from brain tumor biopsies, indicating the feasibility of this approach for such heterogeneous tumors^15^. The measurement of gene expression in single cells however has a number of experimental pitfalls such as the so-called “dropout event” or lack of detection of some RNAs^16,17^. More worryingly, highly plastic cells such as microglia or macrophages are expected to exhibit temporal fluctuation in gene expression. This transcriptional burst is covered in the transcriptome analysis of cell populations but not in a single cell analysis. As a consequence, different intermediate transcriptional states of TAMs - that would be part of a longitudinal and regional TAM signature - will be lost in a single cell analysis and hidden in the analysis of a large number of cells but will be kept in the analysis of a small number of cells. These intermediate states potentially represent relevant therapeutic targets, making the analysis of a limited number of reactive cells -such as cells of the immune system- more relevant than that of individual cells. The limitations and reliability of this approach have not been systematically determined yet. Reducing cell numbers might increase the variability in gene expression according to the law of large numbers. The response of highly plastic cells to the same external stimuli might as well differ according to their number. Thus, what is the minimum number of cells that we can analyze and that will lead to results that are not significantly different from those obtained with a more standard number of cells?

To answer this question, we investigated how much we can reduce the number of cells without affecting the gene expression profile analyzed by standard procedures that they would exhibit when analyzed at a higher cell number. As a cellular model, we used human primary monocyte-derived macrophages (MDMs). Besides representing the precursors of macrophages that infiltrate tumors, these cells offer the advantages of being easily isolated from various donors (biological variation) and having a well-characterized response to inflammatory stimuli^18,19^. In order to simulate *in vivo* conditions in which macrophages are exposed to multiple pro- and/or anti-inflammatory stimuli, we treated MDMs with two Toll-like receptor (TLR) ligands or with two cytokines to polarize them towards a defined pro- or anti-inflammatory status, respectively. Cells were seeded in decreasing amount but at the same density in multi-well plates with various diameters. Following treatment, the resulting macrophage states were characterized by RT-qPCR. Gene expression levels in cells seeded at the highest cell number were compared with levels in cells seeded at the lower cell numbers. Statistical analyses were carried out to assess the degree of change between the two conditions and to select the lowest possible number of cells that maintains sensitivity of and reliability in the gene expression measurements. Finally, we performed functional enrichment and network analyses with these data as a means to understand the biological processes and molecular interactions that are perturbed under changing conditions.

## Results

### Decreasing the number of cells does not affect the expression level of a selected set of genes

In order to determine the lowest number of cells that enables a reliable detection of gene expression, comparable to that detected in high cell numbers, we first analyzed mRNA levels of a small set of genes in MDMs after 24 h of treatment with lipopolysaccharide (LPS) and polyinosinic-polycytidylic acid (poly(I:C)) (hereinafter referred to as M(LPS/IC)). LPS, a ligand of TLR-4, and poly(I:C), a ligand of TLR-3, are pro-inflammatory molecules that trigger tumoricidal activities of macrophages and TAMs^8,18^. Cells from the same macrophage preparation were seeded at various numbers but at constant density in vessels of decreasing size (see Table 1). The highest number of cells we tested, referred to as standard number of cells, was 100,000 cells seeded in one well of a 6-well plate. The lowest vessel we tested was the well of a 96-well plate in which 3,610 cells were seeded. We did not assay a lower number of cells because it would be impractical for any type of molecular analysis using standard methodologies and equipment.

**Table 1:** Seeding conditions of monocyte-derived macrophages in vessels of different size.

Multiple well plate	Area/well (cm^2^)	Seeded cell number/well	µl medium/well
6 well	8,87	100,000	4,000
12 well	3,90	44,000	1,760
24 well	1,90	21,400	860
48 well	1,00	11,300	450
96 well	0,32	3,610	140

To keep the cell density identical, the cell number per vessel was calculated by dividing the vessel area by the reference vessel area (8,87 cm^2^) and multiplying the result by the standard cell number (100,000).

**Figure 1:**
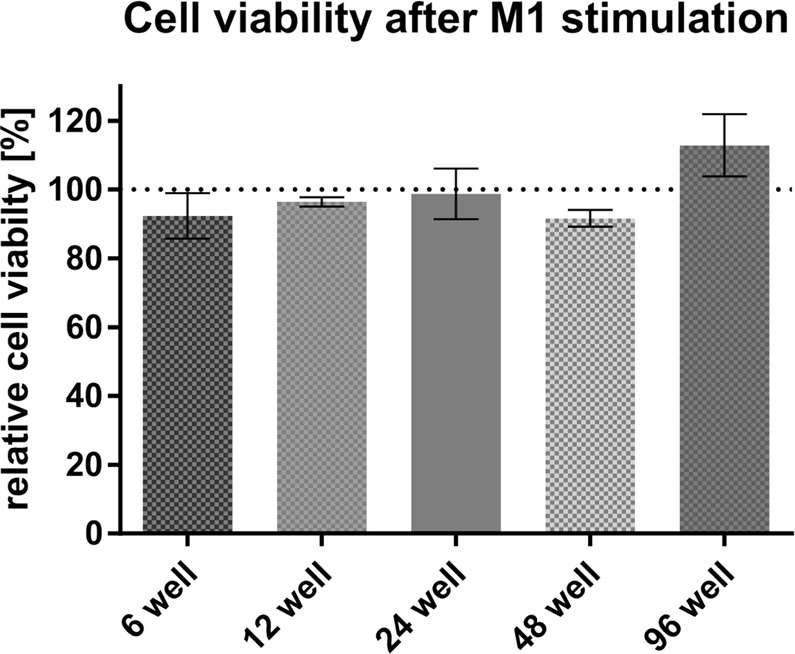
Viability of human MDMs 24 h after treatment with LPS and poly(I:C). Data are expressed as viability of treated cells relative to viability of untreated cells. Values are means ± SD of at least three technical replicates. Statistical analysis was performed using a two-tailed t-test. Calculated p-values: 6 well, untreated vs M1: 0.1683; 12 well, untreated vs M1: 0.6438; 24 well, untreated vs M1: 0.8825; 48 well, untreated vs M1: 0.1370; 96 well, untreated vs M1: 0.1180.

**Figure 2:**
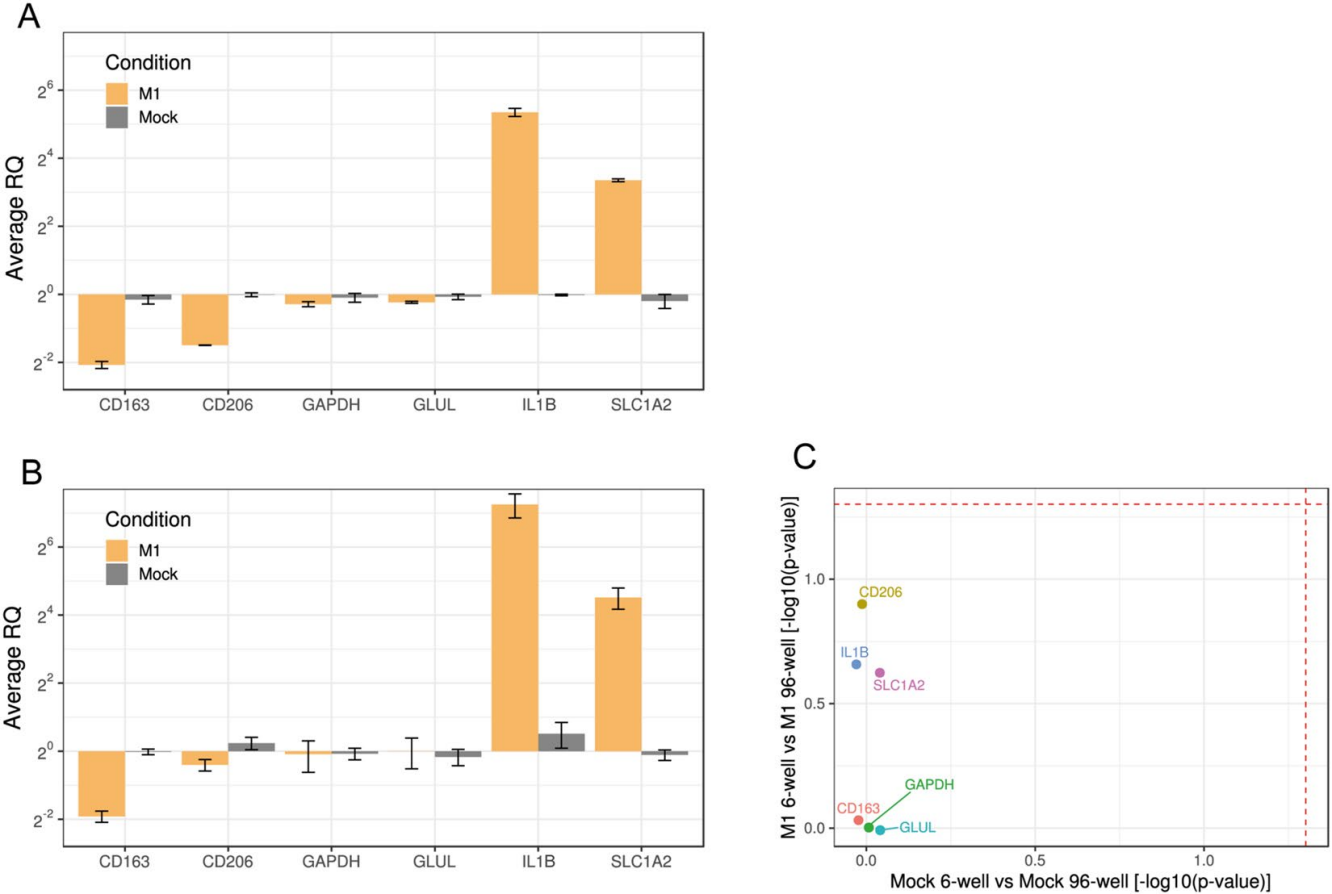
Relative gene expression of human MDMs seeded in 6-well or 96-well plates, 24 h after M(LPS/IC) stimulation (M1) or absence of stimulation (Mock). (**A**) qPCR analysis of 6-well samples. (**B**) qPCR analysis of 96-well samples. M1 values are relative to mock which is normalized to 1. Average RQs of three technical replicates ± SD are shown. Technical replicates: individual wells of a 6-well plate, pool of two wells of a 96-well plate. Reference genes: *SDHA*, *HPRT1*. (**C**) Scatter plot showing the statistical comparison between seeding conditions for matched treatments. Dashed lines correspond to the significance level ɑ = 0.05.

We first assessed whether the applied stimulus affects cell viability. As shown in Fig. 1, there were no significant changes in cell viability after 24 h of stimulation at any seeded cell number. We next analyzed and compared the expression of a set of 6 genes in cells seeded at the standard number (6-well plate) and at the lowest number (96-well plate). RNA extraction and RT-qPCR were performed with technical replicates consisting of individual wells of the 6-well plates and a pool of two wells of the 96-well plates. After 24 h of pro-inflammatory treatment with the LPS/poly(I:C) combination, cells seeded at the highest number (100,000 cells per well in 6-well plates) exhibited the expected profile. Gene expression of *IL1B* and *SLC1A2* was upregulated^18,20^, that of *CD163* and *CD206* was downregulated^18,21^, whereas that of *GAPDH* (our unpublished observations) and *GLUL*^22^ were not altered (Fig. 2A). An expression profile similar to that of the standard condition was observed for cells seeded at the lowest number (Fig. 2B). The statistical analysis of these data (see the Materials and Methods) did not show significant differences between the standard condition and the lowest cell number (Fig. 2C). From these results we concluded that it is possible to decrease the number of cultured cells to a minimum of 3,610 cells per well of a 96-well plate without inducing significant changes in the expression of a panel of genes. In the next experiments, gene expression levels were analyzed and compared in cells seeded in the standard condition (6-well plates) and cells seeded at the lowest number (96-well plates).

### Different stimuli induce the expected gene expression profile independently of cell number

**Figure 3:**
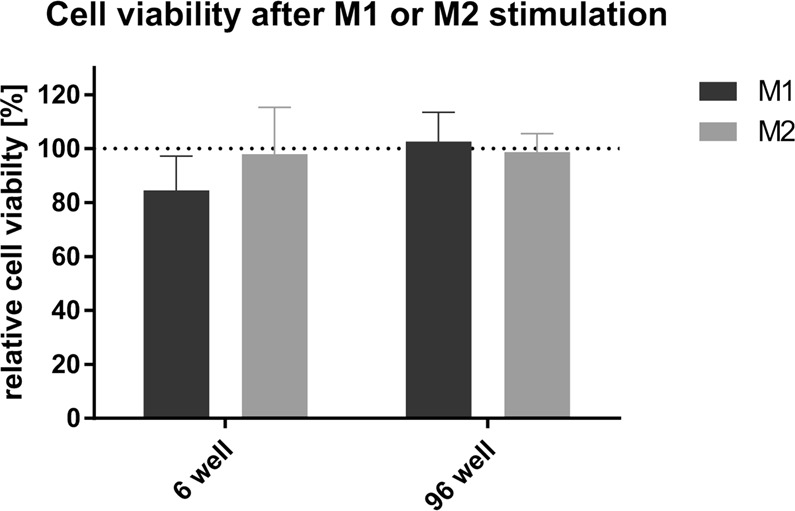
Cell viability of human MDMs 24 h after stimulation with LPS and poly(I:C) (black) or IL-4 and IL-10 (grey). All values are means ± SD of at least three independent experiments. Statistical analysis was performed using the Kruskal-Wallis method. Comparison of M(LPS/IC) vs M(IL-4/IL-10) vs untreated cells revealed no significant differences.

**Figure 4:**
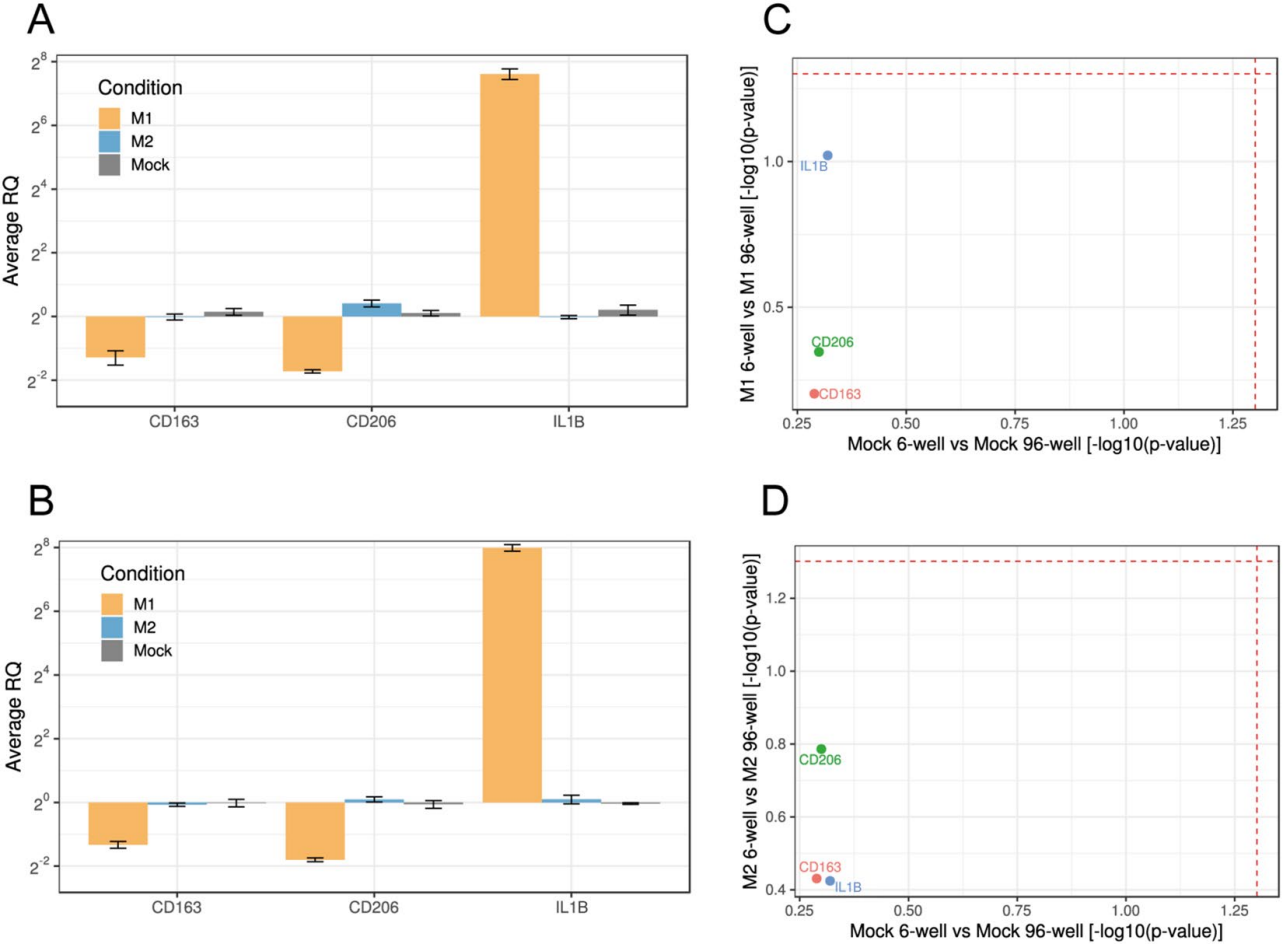
Relative gene expression of human MDMs seeded in 6-well or 96-well plates, 24 h after stimulation with LPS and poly(I:C) (M1), IL4 and IL-10 (M2) or absence of stimulation (Mock). (**A**) qPCR analysis of 6-well samples. (**B**) qPCR analysis of 96-well samples. M1 and M2 values are relative to mock which is normalized to 1. Average RQs of three technical replicates ± SD are shown. Technical replicates: individual wells of a 6-well plate, pool of two wells of a 96-well plate. Reference genes: *SDHA*, *HPRT1*. (**C,D**) Scatter plots showing the statistical comparison between seeding conditions for matched treatments (C = M1, D = M2). Dashed lines correspond to the significance level ɑ = 0.05.

We next analyzed gene expression in MDMs treated with an anti-inflammatory stimulus and compared it with the MDM response to the pro-inflammatory stimulus. These stimuli are expected to induce distinct pro-inflammatory and anti-inflammatory phenotypes that should translate into the opposite expression profile of the inflammatory genes *IL1B*, *CD163* and *CD206*^18,21^. Cells from individual preparations of MDMs were seeded in 6- and 96-well plates and were left untreated or were incubated for 24 h with the pro-inflammatory M(LPS/IC) stimulus or with the anti-inflammatory combination of the interleukin-4 (IL-4) and interleukin-10 (IL-10) (M(IL-4/IL-10)) stimuli. After having excluded possible cytotoxic effects of the M(IL-4/IL-10) stimuli (Fig. 3), gene expression was analyzed by RT-qPCR. Consistent with our observations (see Fig. 2), M(LPS/IC) stimulation induced the expected pro-inflammatory profile. That is, upregulation of the pro-inflammatory *IL1B* and downregulation of the anti-inflammatory *CD163* and *CD206*, both in MDMs seeded at the standard and at the lowest number of cells (Fig. 4). M(IL-4/IL-10) stimulation did not alter the level of *IL1B* or *CD163* expressed by untreated macrophages, suggesting a basal anti-inflammatory status of these cultured MDMs for which the expression of these genes could not be modulated further by the IL-4/IL-10 stimulus. It did, however, increase the level of *CD206* expression, indicating that MDMs were responsive to the anti-inflammatory stimulus. This expression profile was observed both for cells seeded at the standard and lower numbers (Fig. 4A,B). The statistical analysis of these data did not show significant differences between the standard condition and the lower cell numbers (Fig. 4C,D). These results confirm that it is possible to decrease the number of cultured cells to a minimum of 3,610 cells without inducing significant changes in gene expression, independently of the type of stimulus to which the cells were subjected.

### Analysis of a larger set of genes confirms the similarity of the gene expression profiles in standard and low numbers of MDMs

**Figure 5:**
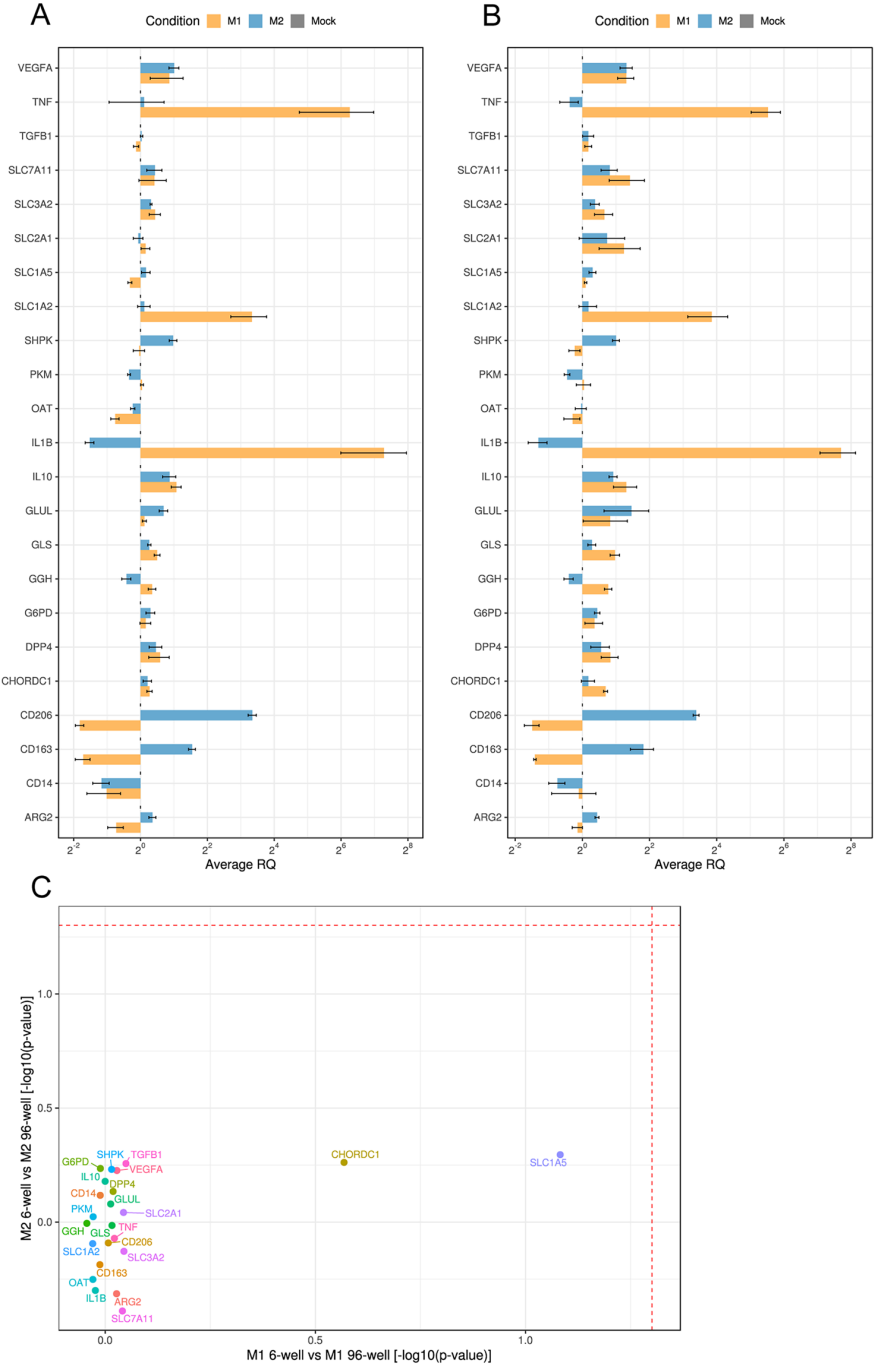
Relative gene expression of human MDMs seeded in 6-well and 96-well plates, 24 h after M(LPS/IC) stimulation (M1), M(IL-4/IL-10) stimulation (M2), or absence of stimulation (Mock). (**A**) Average RQ values of 6-well samples. (**B**) Average RQ values of 96-well samples. M1 and M2 values are relative to mock which is normalized to 1. Bars represent means of monoplicates of 3 independent experiments ± SEM. Technical replicates: individual wells of a 6-well plate, pool of thirty-two wells of a 96-well plate. Reference genes: *SDHA*, *18S*, *GAPDH*. (**C**) Scatter plot showing the statistical comparison between seeding conditions for matched treatments in M(LPS/IC) and M(IL-4/IL-10) stimulated macrophages. Dashed lines correspond to the significance level ɑ = 0.05.

To further demonstrate that the gene expression profile detected in the low number of cells is similar to the profile detected in a larger number of the same cells, we extended our analysis to a broader spectrum of 28 genes. We included genes whose expression is regulated during inflammation and that code for: proteins involved in the immune response (e.g. TGFß, TNF), (metabolic) enzymes (e.g. arginase, dipeptidyl peptidase 4) and metabolite transporters (e.g. EAAT2, xCT). Selection of these genes was based on reported data, as well as on our own unpublished observations (see Table 2 for the complete list of genes). MDMs were seeded in 6-well plates and in 96-well plates and were either left untreated or were treated with the M(LPS/IC) or the M(IL-4/IL-10) stimulus for 24 h. Extracted RNA was then analyzed by quantitative RT-qPCR. Analysis of 28 genes with RT-qPCR requires more RNA than the amount that it is possible to extract from 3,610 cells. This analysis was therefore conducted with technical replicates consisting of a pool of thirty-two wells of the 96-well plates. The robustness of the qPCR was increased by including three instead of two reference genes (*SDHA*, *18 S* and *GAPDH*; see Materials and Methods). With the exception of five genes (*ARG1*, *IFNG*, *IL4*, *IL6*, *IL13*), all the genes listed in Table 2 were detectable in all tested conditions. The same profile was observed in both setups (Fig. 5A,B). The statistical analysis of these data confirmed that there were no significant differences between the two setups (Fig. 5C). These results indicate that we can perform an unbiased evaluation of the expression of a few genes of interest in a low number of cells.

**Table 2:** List of TaqMan® primers used for qPCR.

Target gene (associated protein)	Assay ID
*18s*	Hs99999901_s1
*SDHA* (Succinate dehydrogenase (ubiquinone) flavoprotein subunit, mitochondrial)	Hs00188166_m1
*HPRT1* (Hypoxanthine-guanine phosphoribosyltransferase)	Hs02800695_m1
*IL1B* (Interleukin-1 beta)	Hs01555410_m1
*CD163* (Scavenger receptor cysteine-rich type 1 protein M130)	Hs00174705_m1
*CD206* (Macrophage mannose receptor 1)	Hs00267207_m1
*GAPDH* (Glyceraldehyde-3-phosphate dehydrogenase)	Hs02758991_g1
*GLUL* (Glutamine synthetase)	Hs01013056_g1
*SLC1A2* (Glutamine synthetase)	Hs01102423_m1
*SLC7A11* (Cystine/glutamate transporter)	Hs00921938_m1
*SLC1A5* (Neutral amino acid transporter B(0))	Hs01056542_m1
*SLC3A2* (4F2 cell-surface antigen heavy chain)	Hs00374243_m1
*GLS* (Glutaminase kidney isoform, mitochondrial)	Hs01014020_m1
*GGH* (Gamma-glutamyl hydrolase)	Hs00914163_m1
*OAT* (Ornithine aminotransferase, mitochondrial)	Hs00236852_m1
*CHORDC1* (Cysteine and histidine-rich domain-containing protein 1)	Hs00854389_g1
*DPP4* (Dipeptidyl peptidase 4)	Hs00897386_m1
*G6PD* (Glucose-6-phosphate 1-dehydrogenase)	Hs00166169_m1
*ARG1* (Arginase-1)	Hs00163660_m1
*ARG2* (Arginase-2, mitochondrial)	Hs00982833_m1
*IL4* (Interleukin-4)	Hs00174122_m1
*IL6* (Interleukin-6)	Hs00174131_m1
*IL10* (Interleukin-10)	Hs00961622_m1
*IL13* (Interleukin-13)	Hs00174379_m1
*TNF* (Tumor necrosis factor)	Hs00174128_m1
*IFNG* (Interferon gamma)	Hs00989291_m1
*TGFB1* (Transforming growth factor beta-1 proprotein)	Hs00998133_m1
*CD14* (Monocyte differentiation antigen CD14)	Hs02621496_s1
*VEGFA* (Vascular endothelial growth factor A)	Hs00900055_m1
*SLC2A1* (Solute carrier family 2, facilitated glucose transporter member 1)	Hs00892681_m1
*PKM* (Pyruvate kinase PKM)	Hs00761782_s1
*SHPK* (Sedoheptulokinase)	Hs00950008_m1

### Gene ontology and pathway enrichment analysis of dysregulated genes

**Figure 6:**
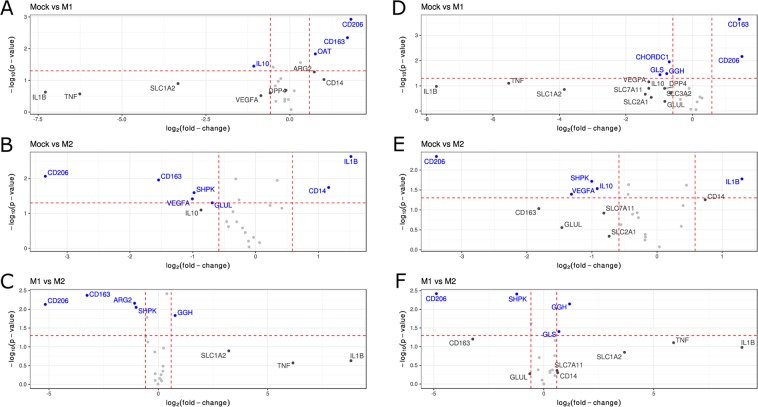
Volcano plots showing the results of the differential gene expression analysis. Only genes that display the strongest alteration in expression in both the 6-well and 96-well setups are indicated by names. Genes whose change in expression was at least 1.5-fold larger than the standard condition and had an associated p-value of at most 0.05 are highlighted in blue. Genes for which only the first condition is met are shown in gray. (**A**–**C**) Data from the 6-well plate analysis. (**D**–**F**) Data from the 96-well plate analysis. Mock = untreated cells, M1 = M(LPS/IC) macrophages, M2 = M(IL-4/IL-10) macrophages.

We finally examined the validity of our approach through a bioinformatics analysis in order to determine and compare pathways altered in treated MDMs seeded at the standard and low numbers. The rationale was that both seeding conditions should result in similar sets of differentially expressed genes and that these genes should be involved in biological processes related to the induced pro- or anti-inflammatory responses. For that purpose, we further analyzed the data generated by the PCR array and determined the genes whose expression showed significant changes after treatment with the M(LPS/IC) and the M(IL-4/IL-10) stimuli. Volcano plots were generated for each differential gene expression analysis (see Materials and Methods): untreated versus M(LPS/IC), untreated versus M(IL-4/IL10) and M(LPS/IC) versus M(IL-4/IL-10), in both setups (Fig. 6). Only genes whose change in expression was at least 1.5-fold larger than the standard condition are indicated by name in Fig. 6. Among those genes, *CD206*, *IL1B*, *SHPK*, *VEGFA* and *GGH* displayed the most robust profile of expression, being identified in each condition and setup. Also note that regardless of the seeding condition (6-well or 96-well plates), the differential gene expression analysis led to similar gene lists.

**Figure 7:**
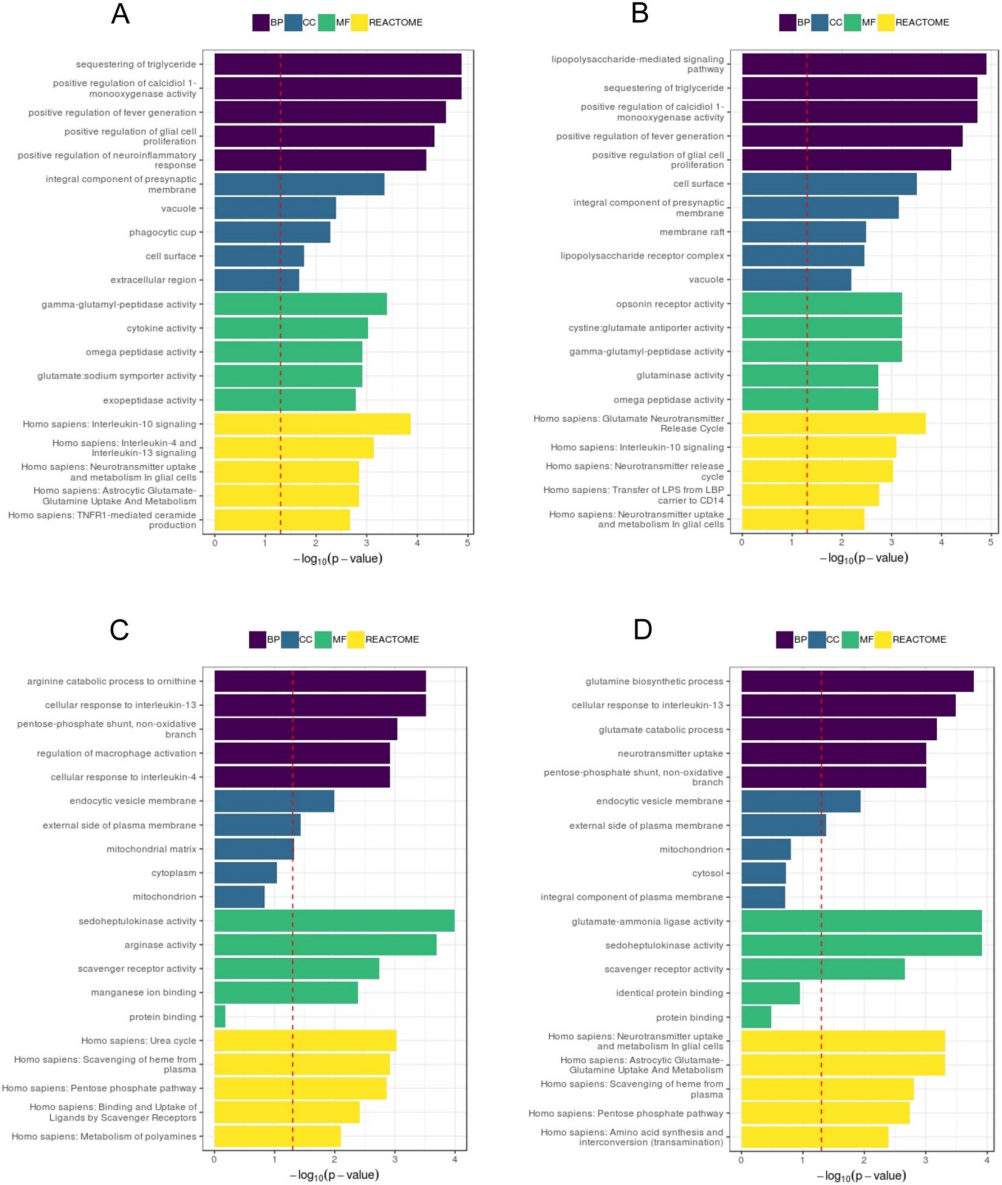
GO and pathway enrichment analysis of data generated from the 6-well and 96-well plate setups. Comparison of the M(LPS/IC) versus M(IL-4/IL-10) macrophages. (**A**) Upregulated terms in the 6-well setup. (**B**) Upregulated terms in the 96-well setup. (**C**) Downregulated terms in the 6-well setup. (**D**) Downregulated terms in the 96-well setup. BP, Biological Process; MF, Molecular Function; CC, Cellular Component.

We then searched for the biological processes and pathways associated with genes differentially expressed in the M(LPS/IC) and M(IL-4/IL-10) macrophages. GO and pathway enrichment analysis using the Reactome database are shown for the 6-well and 96-well plate setups (Fig. 7). Both seeding conditions led to very similar GO terms and Reactome pathways. Indeed, more than 50% of the GO terms and pathways were shared by both conditions whereas other GO terms and pathways presented high similarity. For instance, the upregulated GO terms “positive regulation of neuroinflammatory response” and “lipopolysaccharide-mediated signaling pathway” were found exclusively in the standard condition and the low cell condition, respectively. These two terms obviously refer to the same biological process that is inflammation, which we expect to be induced in M(LPS/IC) macrophages. Similarly, the GO terms “phagocytic cup” and “membrane raft” were found exclusively in the standard condition and the low number of cells condition respectively and refer both to membrane dynamics. These differences are most likely to be explained by the still “low” number of genes we analyzed and would disappear by increasing the number of tested genes. We thus can conclude that, when we use a low number of cells and identify genes of interest, we can trust that the enriched pathways are of value.

**Figure 8:**
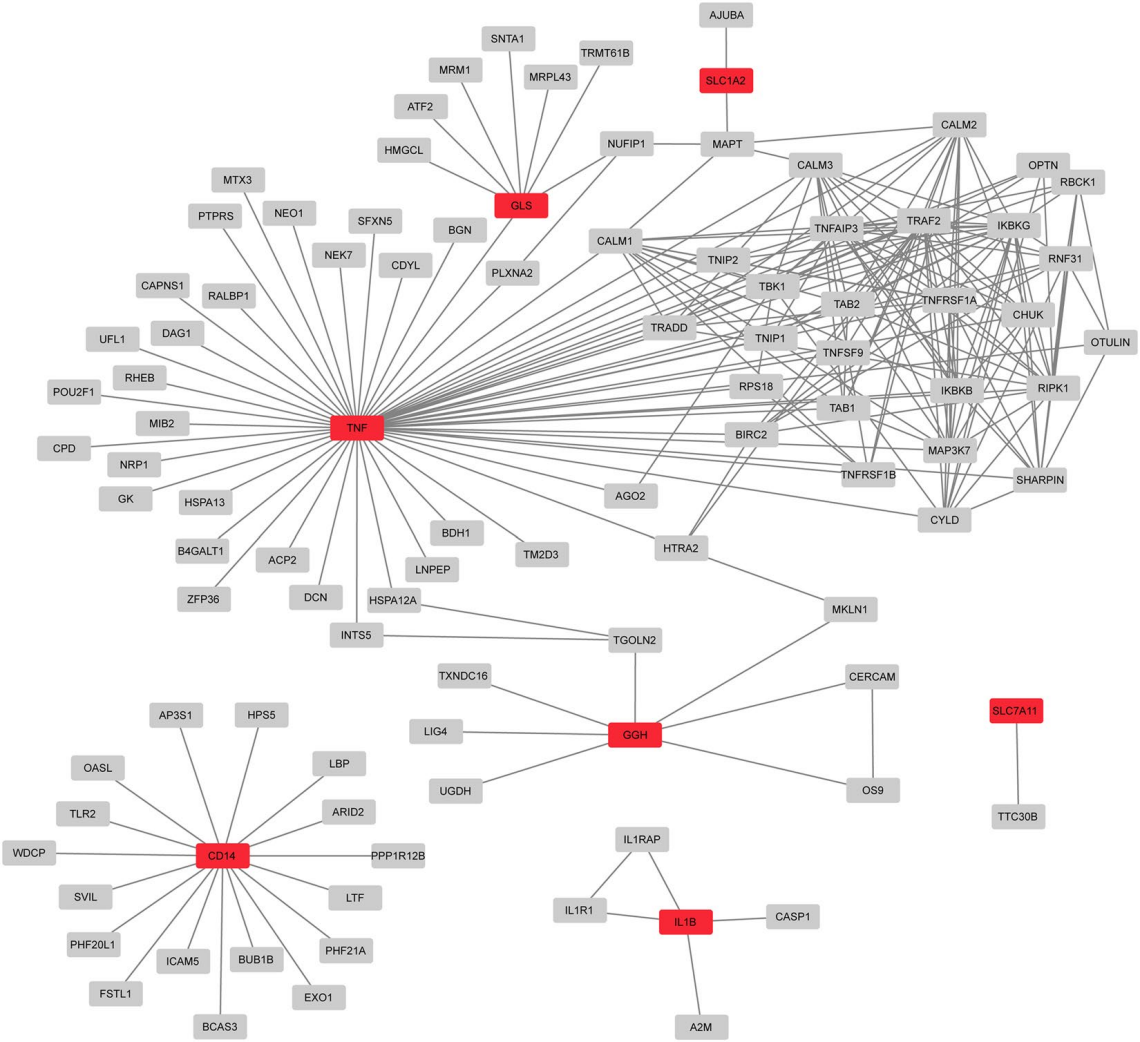
Direct protein-protein interaction partners (gray nodes) of proteins coded by differentially expressed genes (red nodes) according to the M1 vs M2 comparison from the low cell number setup.

The results of the GO and pathway enrichment analysis prompted us to perform a network analysis with genes from the low cell number setup. This included all genes showing at least a 1.5-fold up- or downregulation in the M(LPS/IC) vs M(IL-4/IL-10) stimulated macrophages: *CD206*, *SHPK*, *GLS*, *GGH*, *CD163*, *GLUL*, *SLC7A11*, *CD14*, *SLC1A2*, *TNF*, *IL1B* (see Fig. 6F). Since genes coding for interacting proteins tend to be co-regulated, we examined the protein interaction network around the proteins coded by those dysregulated genes to point to affected pathways. A reference protein-protein interaction network was constructed using high-quality interactions from the HIPPIE database (see Materials and Methods)^23^. Based on this network, subnetworks containing only the proteins coded by the up- or down-regulated genes and their one-level neighbors (i.e. the proteins that directly interact with them) were built (Fig. 8). This network analysis revealed a connection between gamma-glutamyl hydrolase (GGH), which is upregulated after M(LPS/IC) stimulation, and other pro-inflammatory markers such as TNF. The possible relevance of this metabolic enzyme to the inflammatory status and functions of tumor-associated macrophages is discussed below.

## Discussion

In this study, we sought to determine the reliability and the limitations in analyzing low numbers of cells in terms of their gene expression. Indeed, a decrease in cell number could result in an increased variability in gene expression or force changes in their response to external stimuli. We demonstrate that gene expression analysis of a low number of cells is as reliable and informative as the analysis of a larger number of cells. We provide an experimental workflow to assess the reliability of gene expression measurements of a low cell number using RT-qPCR followed by the statistical and computational analysis of the gene expression profiles that we obtained. This experimental workflow is applied to human monocyte-derived macrophages and presents a valid framework for similar studies with other cell types.

We investigated a cellular experimental system that combines different sources of variability: the genetic background of healthy blood donors which is reflected in each of the macrophage preparations used for the study; the stimuli applied to the cells; and finally, the inherent heterogeneity of an *in vitro* culture of primary cells. We kept the seeding density of the cells constant in order to ensure that we would assess the effect of cell number and not that of cell density. Given that our standard number was 100,000 cells/well seeded in a 6-well plate, the size of the smallest vessel available for cell culture, the 96-well plate, imposed a constraint of 3,610 cells on the lowest number of seeded cells. We did not observe statistical differences in the profile of a given set of genes expressed by macrophages from the same preparation and seeded at 3,610 or 100,000 cells. This indicates that, in our experimental system, we can lower the number of macrophages to 3,610 without the risk of introducing an artefactual variability factor. A practical consequence is that more experiments can be conducted with the same batch of cells by using less cells per experiment. In the case of cells isolated from a tissue and that can only be extracted in limited amounts, such as tumor-associated macrophages, our results suggest that 3,610 cells would be sufficient to obtain statistically reliable gene expression measurements. This low number of cells is indeed not meant to be used for the analysis of a large number of parameters (e.g. number of genes) because of the physical limit it imposes on the quantity of material to be analyzed (e.g. RNA). As reported in Figs. 2 and 4, assessing the expression of seven to eight genes (including two reference genes) with our RT-qPCR protocol required pooling material from two wells of cells seeded at the lowest number. Although we *de facto* analyzed the RNA extracted from 7,220 cells, these cells were cultured separately and thus represent cellular replicates. Our study highlights that working with a low cell number certainly imposes a limit on the quantity but not on the quality of the parameters (genes, in this study) analyzed.

The robustness of our analysis was tested by applying two different types of inflammatory stimuli to these very plastic cells that are well characterized for their swift response to any modification of the extracellular milieu. The stimuli we selected trigger different signaling pathways and activate different transcription factors, resulting in two very different molecular and functional profiles of macrophages. The gene expression profiles obtained after the pro- or the anti-inflammatory activation were evaluated using a restricted set of genes. This evaluation was confirmed in the analysis of a larger set of genes and showed the expected changes in the expression levels of specific genes in both setups (standard versus low number of cells). We thus detected the upregulation of *TNF*, *SLC1A2* and *IL1B* after M(LPS/IC) stimulation or the upregulation of *CD163*, *CD206* and *SHPK*^24^ after M(IL4/IL10) stimulation. Moreover, we observed a remarkable similarity in the expression profiles of all genes displayed by both low and standard numbers of cells after the pro- or the anti-inflammatory activation. It thus appears that notwithstanding the use of a very sensitive type of cells (macrophages), the usual sources of technical (such as the batch effect) or biological variation (such as cell heterogeneity or plasticity) neither had an impact on the analysis of the low number of cells we determined nor did they compromise its reproducibility. This leads us to the assumption that this approach could be valid for other types of cells and could be used whenever the cellular material is limited.

Statistical analyses and network analyses with gene expression data of the larger set of genes provided further evidence for the reliability of the data obtained from the low number of cells. Indeed, similar lists of differentially expressed genes and enriched pathways were obtained from both setups. Interestingly, the investigation of dysregulated genes from a protein network perspective suggested interaction between two metabolic enzymes, the gamma-glutamyl hydrolase (GGH) and the glutaminase (GLS), with the pro-inflammatory cytokine TNF, which are all upregulated after M(LPS/IC) stimulation. GGH is a critical enzyme in the regulation of folates. It is responsible for the intracellular cleavage of the poly g-glutamates^25^, releasing glutamate as one reaction product. GLS is involved in glutamate/glutamine metabolism which is highly relevant to the inflammatory status of macrophages^26,27^. Whether and how these proteins interact and what functional meaning it would have for the immunometabolism of macrophages is worth investigating.

In closing, this study has focused on only one cell type, human macrophages, and one target of analysis, mRNA, as a proof of principle. We have used standard RT-qPCR and laboratory equipment to measure gene expression in these very plastic primary cells seeded at high and low cell numbers. The statistical and computational analysis of the gene expression profiles we have obtained in different conditions of cell seeding and treatment prove our hypothesis that the analysis of a low number of cells is not only reliable, but also informative. Having shown the soundness of our approach in this experimental framework, we hope this work will motivate further studies on different types of cells. Indeed, our study is of relevance for a wide range of biologists and biomedical scientists who could adapt the experimental workflow we provide to their own experimental needs and questions.

## Materials and Methods

### Ethics statement

Buffy coats were purchased from the Transfusion Center of the University Medical Center of the Johannes Gutenberg University (Mainz, Germany) and were obtained from anonymized healthy blood donors. All buffy coats used in this study are residual biological materials made available by the Transfusion Center to scientists on a randomized basis. Blood samples are collected and processed in accordance with the relevant German guidelines and regulations. Personal data is neither collected nor shared for this material.

### Monocyte isolation and differentiation into MDMs

Buffy coats were isolated from whole blood of healthy donors collected in CPD bags at the Transfusion Center (University Medical Center of the Johannes Gutenberg University). Briefly, after an initial centrifugation without a density gradient, the pelleted erythrocytes and the top plasma layer were transferred into new bags, leaving the interface (buffy coat) in the original bag. Each unit (approx. 460 ml) of whole blood yielded an approx. 60 ml buffy coat.

Peripheral blood mononuclear cells (PBMC) were isolated from buffy coats as follows. The total volume of each buffy coat was filled up with sterile PBS to a final volume of 120 ml. Afterwards, 10 ml Ficoll®-Paque PREMIUM 1.073 (GE Healthcare) were overlaid with 30 ml of the diluted blood and centrifuged (40 min, 400 rcf, RT, without brake). The PBMC containing layer was isolated and centrifuged again (5 min, 450 rcf, RT). The cell pellet was resuspended in 10 ml erythrocyte lysis buffer (0.15 mM NH_4_Cl, 10 mM KHCO_3_, 0.1 mM EDTA, pH 7.2–7.4), incubated for 5 min on ice and centrifuged (5 min, 450 rcf, 4 °C). The pellet was resuspended in PBS, centrifuged (15 min, 200 rcf, RT), resuspended again in PBS and centrifuged (10 min, 450 rcf, RT). Finally, the pellet was resuspended in 1x NB complete medium [10 ng/ml basic fibroblast growth factor (PeproTech), 20 ng/ml epidermal growth factor (PeproTech), B27™ supplement (Gibco) in Neurobasal™-A medium (Gibco)] and cells distributed on 10 cm Petri dishes (Sarstedt). After 2.5 h of incubation (37 °C, 5% CO_2_), supernatants were collected and replaced by cDMEM [DMEM (Sigma), 10% heat-inactivated FCS (Sigma), 2 mM L-Glutamine (Gibco), 50 µg/ml Gentamicin (Gibco)] containing 20 ng/ml macrophage-colony stimulating factor (M-CSF, Biolegend) for cell differentiation into MDMs. The collected supernatant was distributed on new 10 cm Petri dishes and incubated overnight (37 °C, 5% CO_2_) for a second round of cell attachment. The next day, supernatants were replaced by cDMEM containing 20 ng/ml M-CSF. All dishes were incubated for one week in cDMEM containing M-CSF and cultured for another week in cDMEM without M-CSF prior to experiments. At the end of these two weeks, cells displayed the typical morphology of macrophages and flow cytometry indicated that more than 94% of the cells expressed the CD11b protein (data not shown). Average yield is 8 × 10^6^–1 × 10^7^macrophages per preparation.

### *In vitro* culture and stimulation

Each independent experiment was conducted with cells from one preparation. Monocyte-derived macrophages were seeded in tissue culture vessels of different size (tissue culture plates, Greiner), adapting cell number to vessel area in order to keep the cell density identical (Table 1). The smallest vessel we could assay was the well of a 96-well plate, hence the lowest number of cells to be tested was 3,610 cells. Cells were seeded in cDMEM and incubated for 24 h (37 °C, 5% CO_2_) to let cells attach and recover from scraping/trypsinisation. Afterwards medium was removed and replaced by low serum-containing cDMEM (1% FCS) supplemented or not with the inflammatory stimuli. Cells were thus left untreated or treated for another 24 h (37 °C, 5% CO_2_) before analysis. Low serum-containing cDMEM was used in order to decrease potential side effects of FCS components during MDMs treatment (e.g. competition with stimuli). Treatment consisted of: 10 µg/ml polyinosinic-polycytidylic acid (InvivoGen) combined with 10 ng/ml lipopolysaccharide (Sigma Aldrich) as pro-inflammatory stimulus; 10 ng/ml Interleukin-4 (BioLegend) combined with 10 ng/ml Interleukin-10 (BioLegend) as anti-inflammatory stimulus. As recommended in^28^, macrophages are described according to the stimuli they were treated with: M(LPS/IC) and M(IL-4/IL-10). For space reasons, M(LPS/IC) and M(IL-4/IL-10) labels appear in graphs and legends as M1 and M2 respectively.

### Determination of cell viability

After 24 h of treatment, PrestoBlue™ Cell viability reagent (ThermoFisher) was added directly to the wells in the culture medium according to manufacturers’ instructions. After 30 min of incubation at 37 °C (5% CO_2_) fluorescence was measured at a multiplate reader (TECAN Infinite® 200 PRO) and cell viability calculated as described in the manufacturers’ protocol.

### Total RNA isolation, cDNA transcription and gene expression profiling

Isolation of total RNA was performed using RNeasy Mini Kit (Qiagen) according to manufacturers’ instructions. After PrestoBlue incubation, cells were washed with 1xPBS and lysed in Buffer RLT (containing 1% ß-mercaptoethanol). All following steps were conducted as described in the manufacturers’ protocol. RNA concentration and quality were determined using a Nanodrop 2200 (ThermoFisher). Only samples showing a 260/280 nm ratio between 1.8 and 2.1 were selected for cDNA transcription which was performed with the Omniscript RT Kit (Qiagen) and random hexamers (Life Technologies). Quantitative PCR (qPCR) analysis was done using TaqMan® primers and a StepOnePlus System (Applied Biosystems). Briefly, for each well of the 96-well qPCR plate (Sarstedt), 10 µl of TaqMan™ Universal PCR Master Mix (ThermoFisher) were mixed with 5 ng cDNA and 1 µl of the appropriate primer (Table 2). All measurements were performed using three technical replicates. The PCR array was conducted with a customized TaqMan® gene expression array plate (ThermoFisher) using the same conditions as mentioned above, without technical replicates. Relative quantification (RQ) of gene expression were determined using the 2^−ΔΔCt^ method^29^. To ensure the robustness of the PCR analyses, we included two reference genes when measurements were performed with technical triplicates (data reported in Figs. 2 and 4) and three reference genes when measurements were performed without technical replicates (data reported in Fig. 5). The reference genes were determined among a set of four candidates (*18S*, *SDHA*, *HPRT1*, *GAPDH*) using the geNorm algorithm^30^. *SDHA* and *HPRT1* were identified as the most stable genes in PCR conducted with technical triplicates. *SDHA* and *18 S*, followed by *GAPDH* were identified as the most stable genes in PCR conducted without technical replicates; *HPRT1* had to be dismissed because of technical issues.

### Statistical comparison between seeding conditions

To compare the RQ of gene expression between seeding in 6- and 96-well plates, we employed a two-tailed t-test under the null hypothesis that there were no differences in expression between conditions. These tests were performed for matched treatments, i.e. mock 6-well vs mock 96-well, M1 6-well vs M1 96-well and M2 6-well vs M2 96-well. The resulting p-values were corrected for multiple comparisons using the Benjamini-Hochberg method.

### Differential gene expression, functional enrichment and network analyses

We identified differentially expressed genes between treatments (mock vs M1, mock vs M2, M1 vs M2) using log2-fold changes accompanied by p-values computed via t-tests. This was done separately for the 6- and 96-well seeding conditions. Genes with absolute log2-fold changes ≥1.5 were considered to be up- or down-regulated. These genes were subjected to Gene Ontology (GO) and Reactome pathway enrichment analyses using the R package FunEnrich^31^. In addition, we constructed a protein-protein interaction network with the direct interactors of the genes showing at least a 1.5-fold up- or down-regulation in the M1 vs M2 stimulated macrophages. For this, we used experimentally validated protein-protein interaction data from version 2.2 of the Human Integrated Protein-Protein Interaction rEference (HIPPIE)^23^. Only interactions with confidence scores above the upper quartile of the score distribution were considered.

## Data Availability

The datasets used and/or analyzed during the current study are available from the corresponding author on reasonable request.
